# Partial Molar Pregnancy Presenting as a Tubal Ectopic Pregnancy

**DOI:** 10.1155/2022/7414190

**Published:** 2022-07-05

**Authors:** Mariam Ayyash, Monica Kole, Quoc Le, Yulei Shen, Monique Swain

**Affiliations:** ^1^Department of Obstetrics and Gynecology, Henry Ford Health, Detroit, Michigan, USA; ^2^Department of Pathology, Henry Ford Health, Detroit, Michigan, USA

## Abstract

**Background:**

Tubal molar pregnancy is extremely rare, with no more than 200 cases reported in the literature. The incidence is approximated at 1.5 per 1,000,000 pregnancies.

**Case:**

We report the case of a 22-year-old woman with an overall initial stable clinical presentation who was noted to have a ruptured ectopic pregnancy. She was surgically treated, and pathology revealed partial hydatidiform molar ectopic pregnancy. At the time of surgical intervention, the treating physicians had not considered molar ectopic pregnancy within the differential diagnosis, since this is a very rare presentation. Once the pathology was discovered, the patient was contacted to be scheduled for close follow-up and counseling to reduce progression to choriocarcinomas.

**Conclusion:**

This case report highlights the importance of sending, reviewing, and following up on pathologic specimens for all patients undergoing surgical intervention for presumed ectopic pregnancy and ensuring that appropriate follow-up is in place for those patients.

## 1. Introduction

The incidence of ectopic pregnancy in the United States has been approximated at 20 per 1000 pregnancies [1]. The incidence of partial or complete molar pregnancy is 1 in 1000 [2, 3]. The incidence of having both an ectopic pregnancy that is also a molar pregnancy is extremely rare. In fact, there are approximately 200 case reports for tubal molar pregnancies reported in the literature with an incidence estimated to be 1.5 per 1,000,000 pregnancies [4]. The majority of these reports have shown a good prognosis following laparoscopic salpingectomy [5–9]. The follow-up differs after a normal ectopic pregnancy compared to a molar ectopic pregnancy [5–9]. That is mainly since molar pregnancies, if left untreated, have the potential to become persistent gestational trophoblastic disease and ultimately lead to malignancy and adverse outcomes. In this report, we present the case of a 22-year-old pregnant woman who presented to the emergency department with mild abdominal pain and was subsequently diagnosed with an ectopic partial hydatidiform molar pregnancy.

## 2. Case

The patient was a 22-year-old, gravida 2, para 1001 who presented to the emergency department with abdominal pain. Her pain had started the previous day, but she attributed it to food poisoning at that time, so she ignored it. At the start of the abdominal pain, the patient reported having nausea and vomiting, but upon presentation to the emergency department, the nausea and vomiting had resolved. She continued to have mild, intermittent abdominal pain similar to menstrual cramps. She also reported having vaginal bleeding similar to menstrual bleeding, and that she had been on her menstrual cycle for the past week. She was tolerating a regular diet and was able to ambulate without difficulties. She reported no associated fever, chills, shortness of breath, or chest pain.

**Figure 1:**
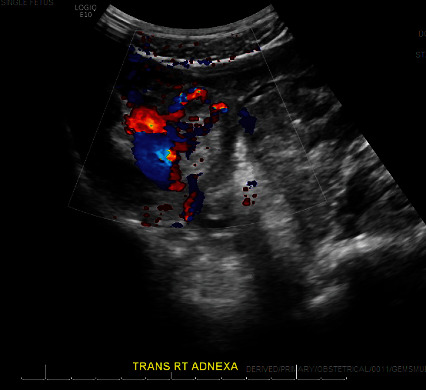
Transvaginal ultrasound of right adnexa. RT: right; TRANS: transverse.

The patient indicated that she did not have any significant past medical history and was not on any medications. Her gynecological history showed past infections with *Trichomonas vaginalis*, *Chlamydia trachomatis*, and *Neisseria gonorrhoeae*, and she tested positive for all 3 pathogens on this emergency department presentation. Her exam revealed minimal vaginal bleeding and was remarkable for right adnexal tenderness. Bedside ultrasound showed free fluid in the right pelvic region, and the ovaries were not visualized. She had stable vital signs and mild leukocytosis with white blood cell count of 15.8 K/uL and platelets of 458 K/uL. Hemoglobin levels were within reference range at 11.6 g/dL. Her *β*-hCG was 23,833 mIU/mL. Rh immunoglobulin was not indicated due to the patient's positive Rh status. Given her overall stable clinical status, we awaited official ultrasound before proceeding with treatment. In the meantime, the patient was given 2 g metronidazole, 1 g azithromycin, and 500 mg ceftriaxone for treatment of the sexually transmitted infections. The official ultrasound showed a normal left ovary, no intrauterine pregnancy, a possible right ovary, and complex fluid within the right adnexa with an echogenic structure measuring 1.16 × 1.20 × 1.1 cm with circumferential color Doppler flow imaging, consistent with the “ring of fire” sign (Figure 1). The findings were concerning for a ruptured right ectopic pregnancy. Subsequently, she was prepared for a diagnostic laparoscopy, removal of the ectopic pregnancy, possible salpingectomy, possible oophorectomy, possible laparotomy, and all other indicated procedures.

**Figure 2:**
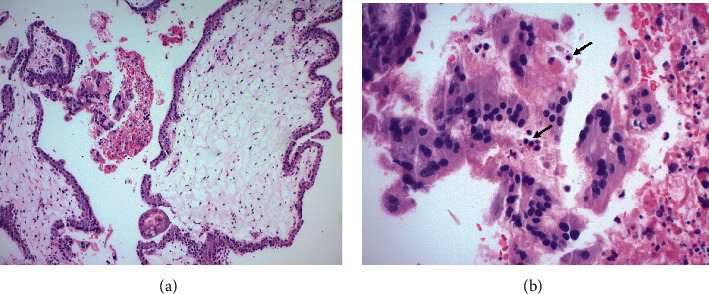
(a) Low power image of a partial mole shows an admixture of normal-appearing and enlarged hydropic villi scalloped borders. (b) High power image shows nucleated red blood cells (arrow) ((a) original magnification ×100; (b) original magnification ×400, hematoxylin and eosin (H&E) stain).

**Figure 3:**
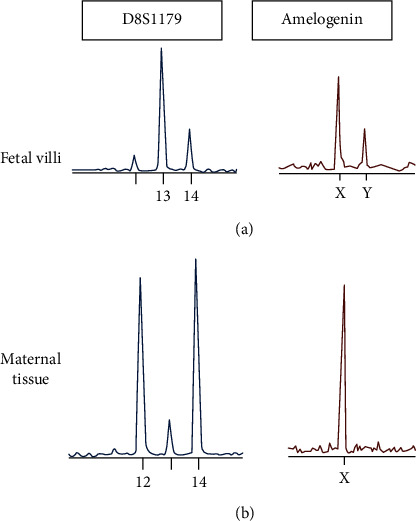
Genetic profiles of a dispermic partial hydatidiform mole by multiplex PCR assay. Dispermic paternal alleles (with homozygous paternal alleles in duplicate quantity in D8S1179 locus) shown, in addition to the presence of one maternal allele. Normal biallelic profiles seen in the (b) maternal endometrium.

The procedure was uncomplicated and revealed 100 mL of hemoperitoneum upon entry. A 3 cm right tubal ectopic pregnancy with evidence of rupture was noted. The patient's bowel and right adnexa were densely adherent to posterior cul-de-sac. The right and left ovaries were buried beneath adhesions and not identified. The left tube was normal appearing but with some adhesions to the left pelvic side wall. Resection of the right fallopian tube with the ectopic pregnancy was performed in a standard fashion by using bipolar energy device to cut and coagulate along the antimesenteric edge of the fallopian tube beneath the portion containing the ectopic and transecting at the cornual end, and the sample was sent to pathology for analysis. The pathology results showed histologic features consistent with partial hydatidiform mole (Figure 2). Molecular studies for gestational disease profile subsequently showed chorionic villi tissue genetically identical to maternal tissue in one set alleles and different in two sets of paternal alleles consistent with a partial mole genotype (Figure 3). The patient was contacted multiple times, but she did not respond via telephone and was lost to follow-up until around 2 months after her procedure. She was seen by the gynecologic oncology service team, and she reported having no symptoms at that visit. She had a regular menstrual cycle in the interim. The care team plan was to perform serial *β*-hCG levels weekly until 3 successive *β*-hCG levels are negative. The patient had a repeat *β*-hCG, which was <10 mIU/mL. She was counseled on contraception and on following up with a gynecologist.

## 3. Discussion

Hydatidiform moles are abnormal gestations resulting from abnormal fertilization and are subclassified into either partial molar or complete molar pregnancies [10, 11]. The type of molar pregnancy is dependent on morphology, pathology, and genetic differences. Complete molar pregnancies generally carry the paternal genomic origin, 46 XX, and are due to the fertilization of an empty ovum by 1 or occasionally 2 sperms [12]. In contrast, partial moles arise from dispermic fertilization of a haploid ovum, resulting in a triploid genome: 69 XXX, 69XYY, or 69 XXY [13].

Pathology is the gold standard to diagnose tubal ectopic pregnancies [14]. The majority of the tubal molar pregnancies are managed surgically and are diagnosed incidentally via histopathology. Ectopic pregnancies that have been managed medically with methotrexate may have a small number of unrecognized tubal molar pregnancies. Some studies have suggested that ectopic molar pregnancies are at times over diagnosed because of improper differentiation from nonmolar hydropic pregnancies [15]. Molar pregnancies are characterized by hydropic changes affecting some or all of the placental villi and are accompanied by circumferential proliferation of trophoblasts [9]. It is important to differentiate molar from nonmolar hydropic pregnancies, as the former have the potential to cause persistent trophoblastic disease [9].

The clinical presentation of patients with ectopic molar pregnancies is generally no different than presentation for traditional tubal pregnancies, which includes abdominal pain and vaginal bleeding. This makes the clinical diagnosis for the two conditions indistinguishable and more challenging. The level of *β*-hCG is not useful in making the distinction either [16]. Additionally, the histologic discrimination between molar pregnancies versus hydropic abortion can be a challenge for pathologists. Sebire et al. reported on the overdiagnosis of ectopic molar pregnancies, where only a small percentage of surgical specimens were actually confirmed as ectopic molar pregnancies by expert gynecological pathologists [17]. It is thus critical to complement the clinical and histological features with ploidy evaluation via DNA flow cytometric analysis [9, 15]. It is worth noting, though, that after histological diagnosis, molecular techniques can distinguish partial from complete mole, but they cannot help to distinguish complete mole from hydropic abortion 15, 18. In our case, following the histopathology of the patient's specimen, molecular studies for gestational disease profile were performed to show the allelic distribution and confirm the partial molar pregnancy diagnosis. The hematoxylin and eosin stain slide was examined, and fetal and maternal DNA was enriched by microdissection for polymerase chain reaction analysis.

The timely diagnosis of molar pregnancy (whether complete or partial) is crucial, as such pregnancies have the potential to cause persistent gestational trophoblastic disease [19] which has the potential to evolve into malignancy and may ultimately lead to adverse outcomes if left untreated [20]. Persistent gestational trophoblastic neoplasia can occur in about 15% to 20% of complete molar pregnancies and 1% to 6% of partial molar pregnancies [20, 21]. Due to such risk of malignancy, after the diagnosis of molar pregnancy, patients are normally followed with weekly quantitative *β*-hCG titers until 3 successive *β*-hCG levels are negative. They are then advised to avoid pregnancy for 6 months and are counseled on appropriate contraception options [21, 22].

Our case report emphasizes the importance of histological examination of the products of conception and tissue diagnosis after surgical management for ectopic pregnancies. While this phenomenon is rare, it is important to keep in mind the possibility of a hydatidiform mole in the extrauterine cavity with an ectopic pregnancy presentation. Also, performing molecular studies to differentiate molar from nonmolar hydropic abortions is critical, as proper management differs between the two. Lastly, it is also critical to continue proper follow-up with appropriate counseling, serial *β*-hCG assessment, and appropriate contraception planning to ensure that such incidents of molar ectopic pregnancies do not progress to choriocarcinomas.

## Data Availability

The case report data used to support the findings of this study are included within the article.
